# Supplemental LED inter-lighting compensates for a shortage of light for plant growth and yield under the lack of sunshine

**DOI:** 10.1371/journal.pone.0206592

**Published:** 2018-11-01

**Authors:** Fasil Tadesse Tewolde, Kouta Shiina, Toru Maruo, Michiko Takagaki, Toyoki Kozai, Wataru Yamori

**Affiliations:** 1 Graduate School of Horticulture, Chiba University, Matsudo, Japan; 2 Center for Environment, Health and Field Science, Chiba University, Kashiwa, Japan; 3 Ethiopian Institute of Agricultural Research, Addis Ababa, Ethiopia; 4 JA Zen-Noh, Kashiwa, Japan; 5 Japan Plant Factory Association, Kashiwa, Japan; 6 Department of Biological Sciences, Faculty of Science, the University of Tokyo, Tokyo, Japan; University of Tsukuba, JAPAN

## Abstract

Supplemental lighting can enhance yield when sunlight is limited, as in winter. As the effect of frequent cloudy or rainy days in other seasons on plant growth and yield remains unclear, we investigated the effect on tomato (*Solanum lycopersicum*) and compensation by supplemental LED inter-lighting. Plants were grown under 30% shade cloth on 0%, 40%, or 60% of days. Lower leaves were illuminated with red and blue LED inter-lighting modules from right after first anthesis, or not illuminated. Shading during 40% and 60% of days diminished daily light integral (DLI) by 26% and 40%, respectively, and reduced shoot dry weight by 22.0% and 23.3%, yield by 18.5% and 23.3%, and fruit soluble solids content by 12.3% and 9.3%. In contrast, supplemental inter-lighting improved the light distribution within plants and compensated DLI, and maintained similar yield and soluble solids content in both shade treatments as in the control. These results clearly show that supplemental LED inter-lighting could efficiently compensate for a shortage of light for plant growth, photosynthesis and thus yield under the lack of sunshine.

## Introduction

Valued globally at 58.2 billion USD, tomato is ranked as the 4th most valuable agricultural commodity or crop after rice, wheat, and soybean [1]. Thus, economic benefits may accrue from investigating the effects of low solar radiation as a result of cloud cover on productivity of greenhouse tomatoes. Despite the “global brightening” reported in many places [2], some areas in East Asia still experience dimming characterized by the reduced in shortwave radiation due to the high or even increasing aerosol concentrations, especially in China and downstream in Japan [3]. In the past 50 years the reduction of solar radiation reaching Earth’s surface globally has averaged 0.51 ± 0.05 W m^−2^ per year, equivalent to a reduction of 2.7% per decade [4]. Thus, it’s important to understand whether cloud cover and low solar radiation limit carbon uptake, photosynthesis, and thus crop productivity despite the improvement in carbon gain in terrestrial ecosystems by an increased ratio of diffuse radiation [5].

Single-truss tomato production systems [6, 7] can reduce labor requirements for training, pruning, and harvesting. Moreover, single-truss tomato cultivation systems are superior to multi-truss tomato cultivation systems because they allow multiple cropping, predictable and consistent harvests, and use of moveable benches, and they have the potential for automation [6–11]. However, at high planting density, light becomes a limiting factor in the growth of single-truss tomato plants, as mutual shading prevents light from penetrating into the lower canopy [10]. This problem is exacerbated in winter [12]. Moreover, frequent cloudy or rainy days during spring to early summer could also affect plant growth and productivity. To maintain optimal growth, plants require optimal light, temperature, water, and nutrients. These factors have long been understood as primary determinants of agricultural productivity [13–15]. In greenhouse horticulture, it is relatively easy to control water, nutrients, and temperature, but not irradiation [16, 17]. Plants experience a highly variable light environment over the course of the day, season, and year [18], and are highly sensitive to their light environment [19, 20], as irradiation directly affects photosynthesis, which determines plant growth and yield [18, 21, 22].

In tomato production, the understory leaves have a very low net photosynthetic rate due to both lower incident light and induced senescence [23, 24]. Frantz *et al* [25] found that supplemental light within a cowpea canopy significantly delayed senescence of the interior leaves. In addition, supplying upward lighting from underneath retarded the senescence of outer leaves of lettuce and increased photosynthetic rate, improving total plant growth [26, 27]. Many studies have shown that supplemental lighting above or within the canopy enhanced the yield of tomato plants when sunlight is limited, mainly in winter [28–33]. In greenhouse crop production, the ‘1% rule of thumb’ says that a 1% reduction in the daily light integral (DLI) results in a 1% reduction in yield [34]. This rule was shown to hold for greenhouse crops such as cucumber and sweet pepper throughout the cropping period [35]. A similar relationship was found in tomato during winter and spring, although the yield reduction was slightly lower in summer [36]. Although it has been investigated a relationship between crop yield and DLI seasonally and/or annually, it would be difficult to eliminate differences in other environmental factors (i.e., temperature, VPD and so on) depending on the season and the year. Recurrent cloudy and rainy days in other seasons could reduce plant growth and thus productivity, however, there has been no research on how supplemental lighting can compensate under continuous cloudy conditions.

Crop productivity under limited light conditions can be enhanced by supplementation using fluorescent lamps, high-pressure sodium lamps, metal halide lamps, or LED lamps [28–33]. LEDs are considered a suitable light source for inter-lighting (lighting within plant canopy) because they produce less heat and are therefore less likely to burn leaves than high-pressure sodium lamps [37]. LED inter-lighting module used in this experiment was found to increase temperature by about 1°C [33]. It has also been reported that temperatures above 33 ^o^C must be avoided with most tomato cultivars when aiming to produce fruit [38]. Usually supplemental lighting should be turned off when solar irradiation exceeds a desired set point, which is about 1300 mmol m^-2^ s^-1^ in a greenhouse [39, 12]. Their development has enabled growers to control light spectral qualities by combining various light sources with different waveband emissions [40, 41, 9, 42, 43]. Our objective was to investigate how cloudy or rainy weather affect tomato plant growth and yield and how supplemental LED inter-lighting could compensate the DLI requirement and thus improve plant growth and yield in single-truss tomato production.

## Materials and methods

### 2.1. Plant material and growth conditions

Seeds of tomato (*Solanum lycopersicum* L. ‘Sanbi’) were sown in 128-cell plug trays filled with vermiculite on 23 April 2014. After 2 days in the dark at 26°C, the trays were transferred to a walk-in environment-controlled growth chamber with environmental control unit (Nae Terrace, Mitsubishi Plastics Agri Dream Co., Ltd., Tokyo, Japan), and seedlings were grown as described [33]. After 3 weeks, on 15 May, the seedlings were transplanted at a density of 10 plants per m^2^ in a greenhouse. The greenhouse was equipped with heat pumps and a pad-and-fan system to control day/night temperatures at 26/18°C. Otsuka nutrient solution (Otsuka Chemical Co., Ltd., Osaka) was used with the application schedule, EC, and pH described by [33]. In high-density single-truss tomato cultivation, each plant is allowed to develop only a single fruit truss [9]. To achieve this, the apical meristem of each plant was pinched after first anthesis, leaving two leaves above and three leaves below the truss, and all flowers that set fruits were kept intact. To improve fruit set, fully blooming flowers were sprayed once with 4-chlorophenoxyacetic acid at 15 mg L^−1^ as described [33].

### 2.2. LED inter-lighting

The understory leaves were illuminated with LED modules (Green Power LED inter-lighting module DR/B, Philips, Eindhoven, the Netherlands. The modules combined blue (with a peak at 440 nm) and red light (with a peak at 632 nm) of 1:4 ratio, respectively, with a photosynthetic photon flux density (PPFD) of 220 μmol m−2 s−1 (for more, see [33]). They were positioned on both sides of the aisle at 50 cm from the stems (10 cm from the mid-canopy leaves), at a height of 60 cm above the polystyrene board under which the root system grew. As yield of single-truss tomato plants is positively correlated with the total incident light during the period from anthesis to harvest [7, 31, 42], we applied LED inter-lighting with a 12-h photoperiod from the very first anthesis until harvest in order to maximize yield while minimizing energy costs.

### 2.3. Cloud cover simulation

**Fig 1:**
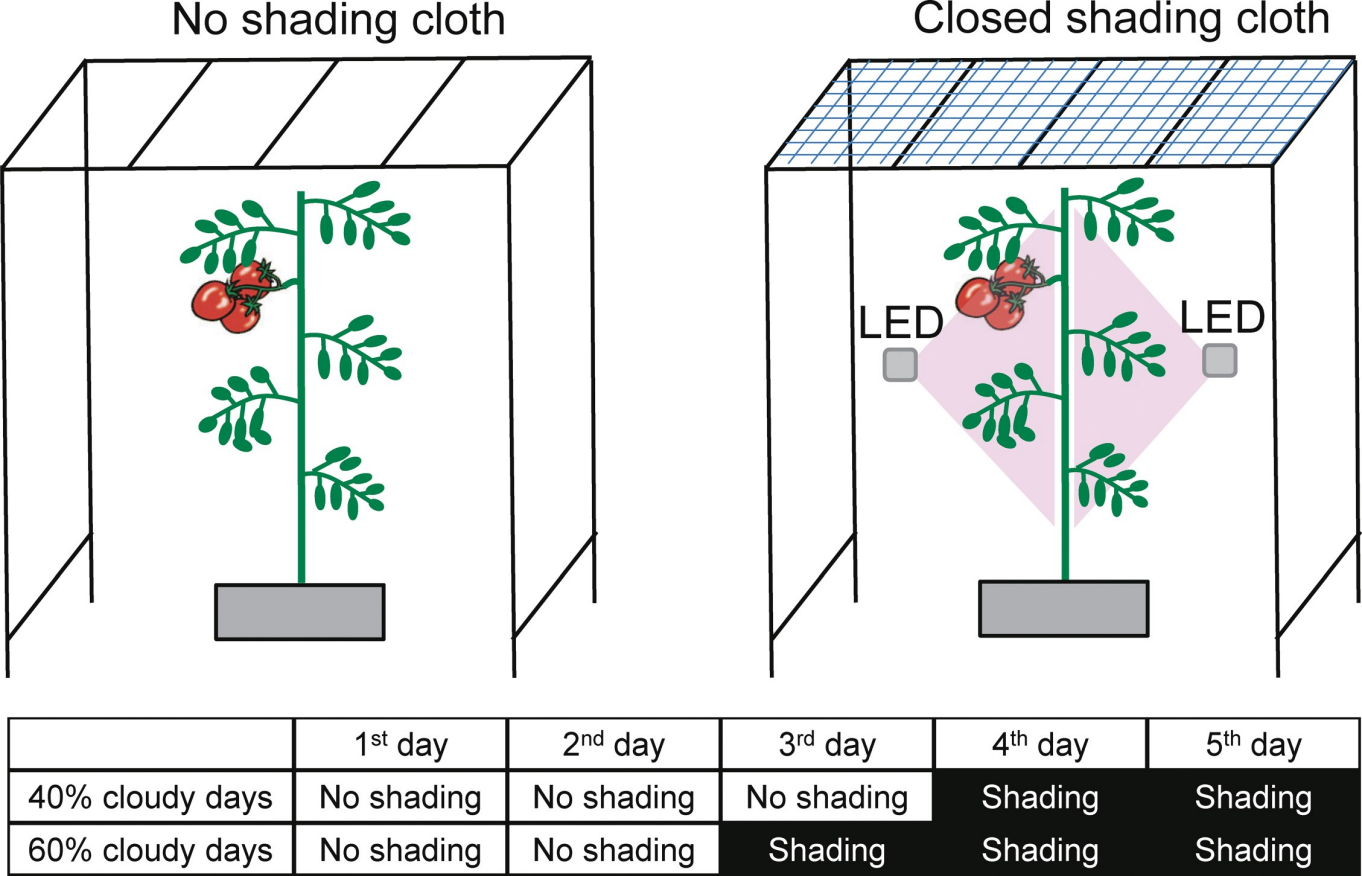
Schematic diagram of experimental schedule and LED inter-lighting used. LED inter-lighting was used when the shade cloth was applied.

Between 40% and 60% of all days over the past 5 years in Japan were cloudy or rainy [44]. To simulate cloudy weather, we used 30% shade cloth to create treatments in which 40% or 60% of days within the growth period were ‘cloudy’ (Fig 1). We used five treatments: 40% and 60% cloudy days with and without supplemental LED inter-lighting, and a control (no shading, no inter-lighting). Each treatment had twenty-five tomato plants, of which fifteen plants were selected randomly and tagged for the measurement of growth and other parameters.

### 2.4. Measurements

#### Plant growth

We measured internode length and stem diameter under the fruit truss, leaf chlorophyll content, leaf area, leaf mass per unit area (LMA), and shoot dry weight. Chlorophyll was determined by chlorophyll meter (SPAD-502 Plus, Konica Minolta, Tokyo, Japan). Leaf area was measured by leaf area meter (LI-3000C, Li-Cor, Lincoln, NE, USA).

#### Light distribution within the plant profile

**Fig 2:**
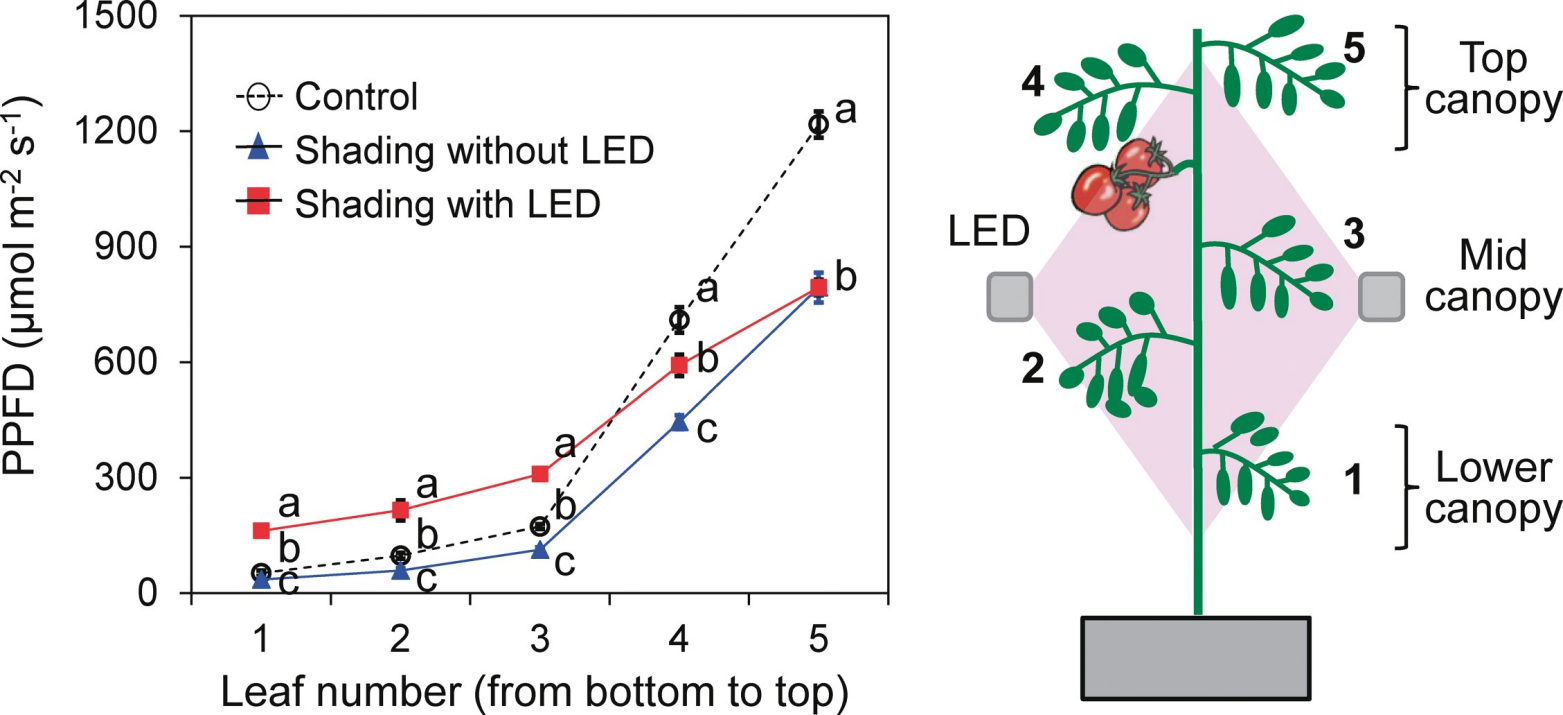
Effects of LED inter-lighting on photosynthetic photon flux density (PPFD) within the plant canopy. PPFD was measured by a quantum sensor held at the same angle as nearby leaves. Data are means ± SEM (*n* = 10). Values marked with the same letter are not significantly different (Tukey’s HSD at *P* < 0.05).

We measured the light intensity at the top (5^th^ leaf), middle (3^rd^ leaf), and bottom (1^st^ leaf) of the canopy (Fig 2) with a quantum sensor (LI-190SA; Li-Cor). The sensor was angled the same as nearby leaves. The LED inter-lighting remained in use. Solar irradiance alone was measured as a control.

#### Daily light integral (DLI)

DLI is an important variable to measure in a greenhouse because it directly influences plant growth, development, yield, and quality. DLI is the amount of photosynthetically active radiation received per day, as a function of light intensity (instantaneous light: μmol m^-2^ s^-1^) and duration (day).

#### Leaf gas exchange

We measured the light-response curve of the photosynthetic rate from 10:00 to 14:00 with a portable gas exchange system (LI-6400; Li-Cor) as described [45–47] in representative leaves from the top of the canopy (5th leaf from the bottom), the middle (3rd leaf from the bottom), and the bottom (1st leaf from the bottom).

#### Yield and fruit quality

We recorded the fresh weight of each fruit and measured two quality parameters [33]: ascorbic acid content, using a reflectometer (RQ Flex Plus, Merck Co., Ltd., Darmstadt, Germany), and total soluble solids content, using a refractometer (Atago 3810 PAL-1, Atago Co., Ltd., Tokyo, Japan).

### 2.5. Statistical analysis

Data were tested in SPSS v. 21.0 software (SPSS, Chicago, IL, USA). The significance of differences between treated plants and controls was analyzed with Tukey’s HSD test. *P* < 0.05 was considered to be significant.

## Results

### Daily light integral (DLI)

Control plants (no shading, no supplemental inter-lighting) received a mean DLI of 8.4 mol m^−2^ (Table 1). Shading of plants for 40% and 60% of days decreased DLI to 6.2 and 5.0 mol m^−2^, respectively, thus decreasing DLI by 2.2 and 3.4 mol m^−2^. Supplemental LED inter-lighting contributed 2.8 and 4.2 mol m^−2^ DLI, respectively. It also increased light intensity and distribution among the middle and lower canopy leaves (Fig 2). In both shading treatments, it increased the light distribution within mid-canopy leaves by 165 μmol m^−2^ s^−1^. The estimated diurnal changes in PPFD indicated that only 30% of the incident light at the top of the canopy reached the middle, and only 15% reached the bottom (Fig 2).

**Table 1:** Effects of shading and LED inter-lighting on daily light integral (mean ± SEM, *n* = 15).

Treatment	Daily light integral (mol m^−2^ d^−1^)	
	Sunlight	LED inter-lighting
Control	8.4 ± 0.7	–
40% shading	6.2 ± 0.9	2.8
60% shading	5.0 ± 0.9	4.2

### Leaf chlorophyll content

**Fig 3:**
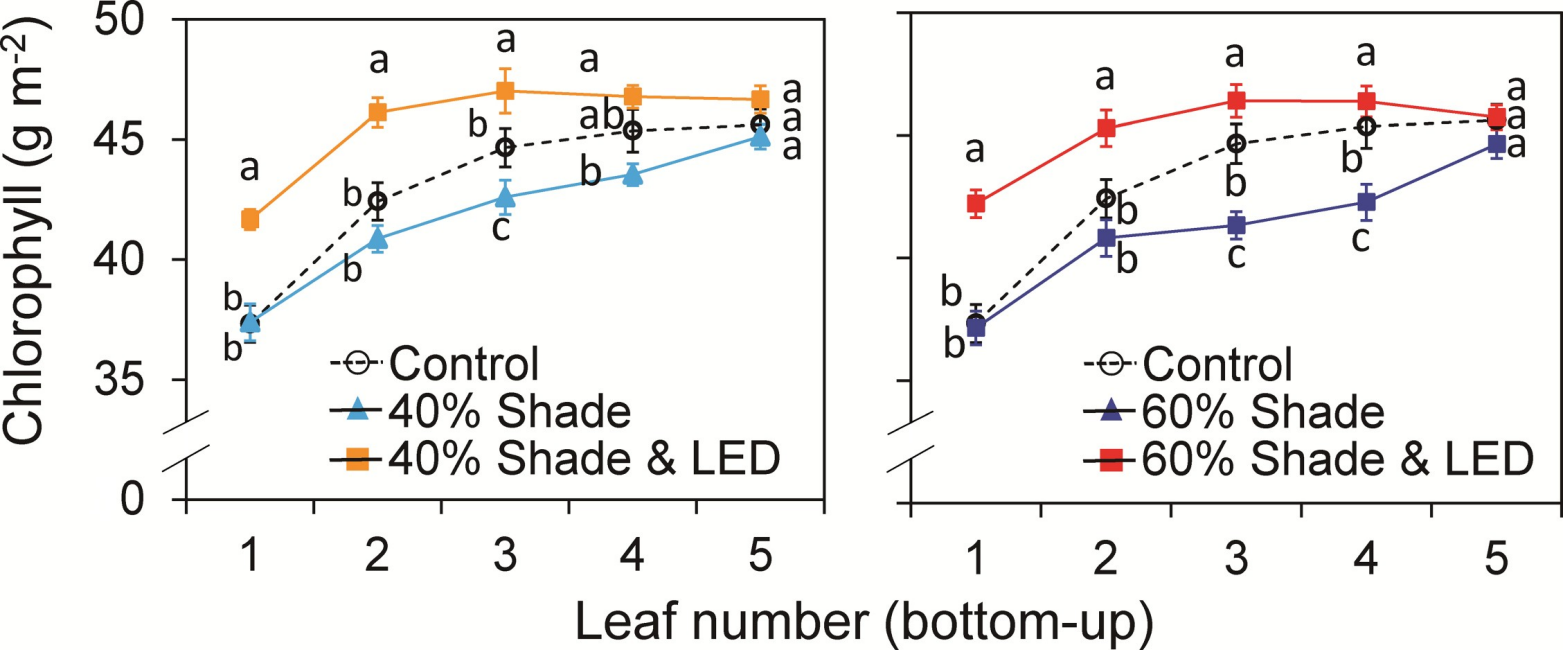
Total chlorophyll contents of single-truss tomato leaves measured in three canopy layers in plants grown under shading during 0%, 40% and 60% of days, with or without LED inter-lighting. Data are means ± SEM (*n* = 15). Values marked with the same letter are not significantly different for the same leaf number in different treatments (Tukey’s HSD at *P* < 0.05).

Leaves in the middle and lower canopy had significantly lower chlorophyll contents than leaves at the top. In the control, the content increased from 37.3 g m^−2^ in the bottom leaf to 45.6 g m^−2^ in the top leaf (Fig 3). In the shading treatments, it decreased further in the mid-canopy leaves, by 5% in the 40% treatment and by 7% in the 60% treatment, but did not differ significantly from the control in the top and lower canopy in either shading treatment (Fig 3). Supplemental LED inter-lighting increased the light distribution within the plants (Fig 2). It overcompensated the chlorophyll content of the mid-canopy leaves in both treatments increased it by 12% in the 40% treatment and by 13% in the 60% treatment of the lower canopy, but had no effect in the top canopy (Fig 3).

### Photosynthesis

**Fig 4:**
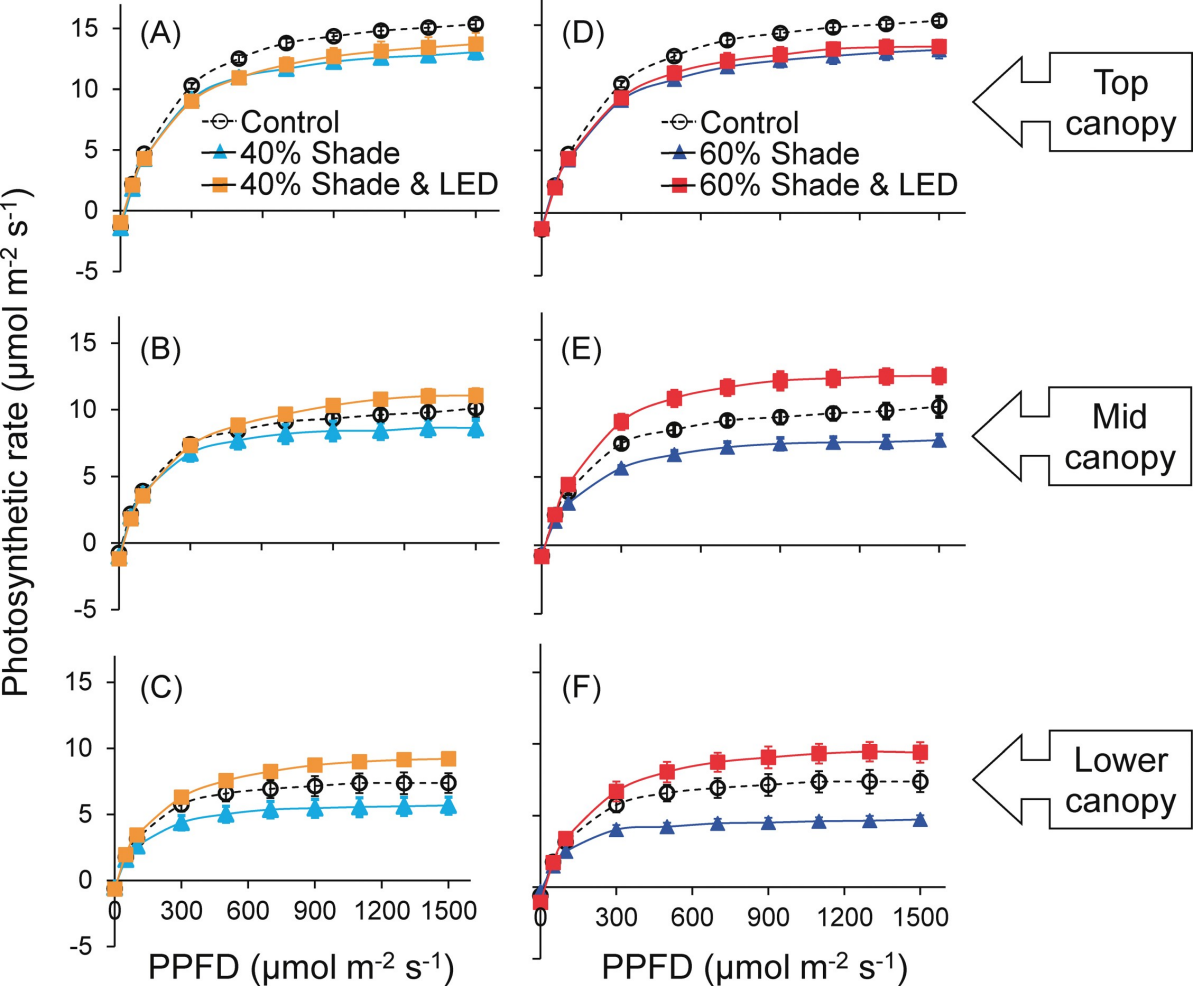
Effects of LED inter-lighting on leaf photosynthetic capacity of single-truss tomato plants. Light-response curve was measured from 10:00 to 14:00 in representative leaves in three canopy layers in plants grown under shading during 0%, 40% and 60% of days with or without LED inter-lighting. Data are means ± SEM (*n* = 5).

Both shading treatments did not affect photosynthetic rate in the mid-canopy leaves at lower light intensity (< 100 μmol m^−2^ s^−1^), but significantly reduced at higher light intensity (> 200 μmol m^−2^ s^−1^) (Fig 4, S1 Table). The photosynthetic rate in the mid-canopy leaves was reduced by 12% at PPFD = 1000 μmol m^−2^ s^−1^ in the 40% treatment, and by 22% at PPFD = 1000 μmol m^−2^ s^−1^ in the 60% treatment. On the other hand, the supplemental LED inter-lighting compensated for the reduction of photosynthetic rate by both shading treatments. The effect in the lower canopy was similar, but the top canopy showed no difference from the control.

### Plant growth

**Fig 5:**
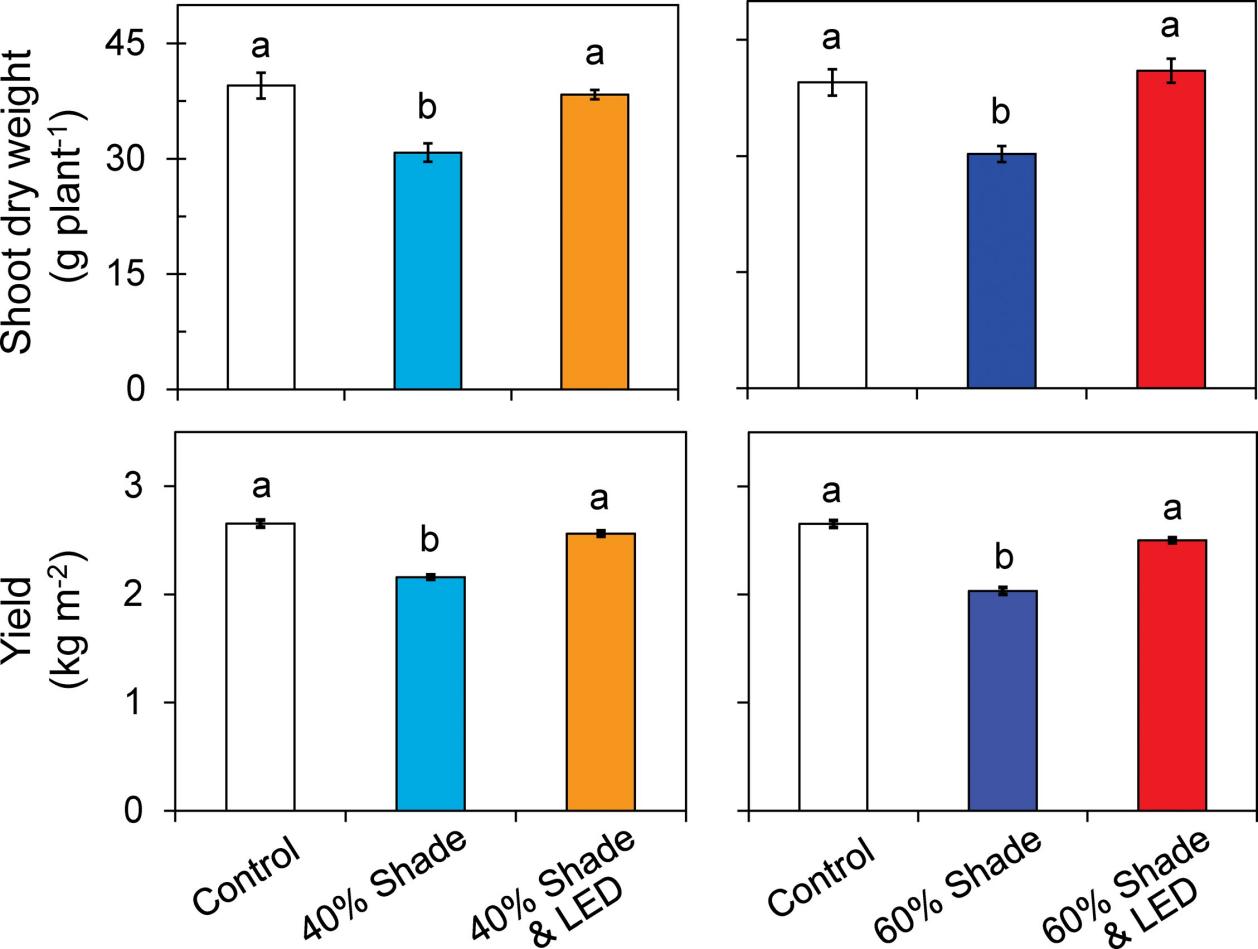
Shoot dry weights and fruit yield of single-truss tomato plants grown under shading during 0%, 40% and 60% of days with or without LED inter-lighting. Data are means ± SEM (*n* = 5). Values marked with the same letter are not significantly different (Tukey’s HSD at *P* < 0.05).

Shading on 60% of days reduced stem diameter by 11.3% and truss diameter by 14.6%, but supplemental LED inter-lighting reversed these differences (Table 2). Inter-lighting increased leaf mass per unit area (LMA) significantly but had no significant effect on internode length or leaf area index (LAI) (Table 2). Shading reduced shoot dry weight by 22.0%–23.3%, but inter-lighting increased it by 19.1%–19.5% to levels similar to the control (Fig 5).

**Table 2:** Effect of shading and LED inter-lighting on growth of single-truss tomato plants (means ± SEM, *n* = 15).

Treatment	Growth parameters				
	Stem diameter (mm)	Truss diameter (mm)	Internode length (mm)	Leaf are index	Leaf mass per unit area
Control	11.5 ± 0.2a	4.8 ± 0.1a	82.7 ± 2.4a	3.3 ± 0.3a	13.6 ± 1.8a
40% shading	10.6 ± 0.2a	4.4 ± 0.1a	84.8 ± 2.3a	3.1 ± 0.2a	11.1 ± 0.5b
40% shading with LED	11.4 ± 0.4a	4.5 ± 0.2a	81.5 ± 3.5a	3.6 ± 0.3a	12.6 ± 1.5a
60% shading	10.2 ± 0.3b	4.1 ± 0.1b	95.6 ± 3.3a	2.6 ± 0.1a	10.9 ± 0.9b
60% shading with LED	10.9 ± 0.2a	4.5 ± 0.2a	93.8 ± 1.9a	3.1 ± 0.3a	13.7 ± 1.1a

Stem diameter and internode length were measured just under the fruit truss. Values followed by the same letter are not significantly different by Tukey’s HSD test (*P* < 0.05).

### Fruit yield and quality

**Fig 6:**
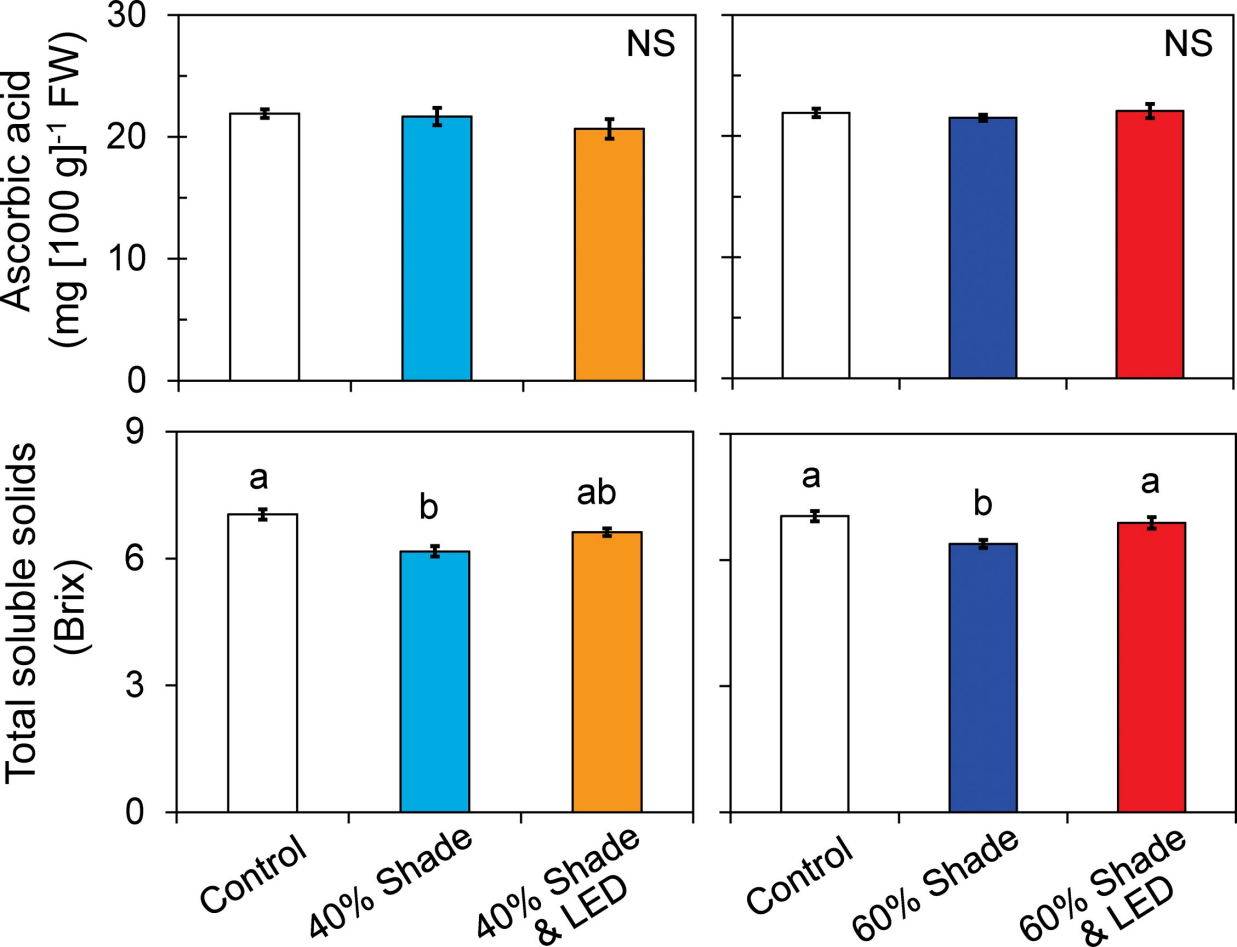
Ascorbic acid and total soluble solids contents of single-truss tomato plants grown under shading during 0%, 40% and 60% of days with or without LED inter-lighting. Data are means ± SEM (*n* = 10). Bars with the same letter are not significantly different (Tukey’s HSD at *P* < 0.05). NS, no significant differences.

Shading decreased fruit yield by 18.5% in the 40% treatment and by 23.3% in the 60% treatment. Thus, a 1% reduction in DLI reduced yield by 0.7% in the 40% treatment and by 0.6% in the 60% treatment. However, supplemental LED inter-lighting compensated for the DLI requirement, improving plant growth and yield to the control levels (Fig 6). Shading also reduced the fruit soluble solids content, by 12.3% and 9.3%, respectively. However, inter-lighting completely compensated it (Fig 6). On the other hand, shading showed no significant effect on fruit ascorbic acid content.

## Discussion

### Supplemental LED inter-lighting enhanced daily light integral and vertical light distribution

Cloud-free weather is infrequent during spring and early summer in temperate regions such as Japan [48] and southern China [49]. Light is further reduced by the greenhouse glazing and structures [17]. In our experiment, shading during 40% and 60% of days diminished DLI by 26% and 40%, respectively (Table 1). A minimum of 4 mol m^−2^ d^−1^ is needed for single-truss tomato production [39]. Since our results show that supplemental LED inter-lighting increased DLI by 2.8 mol m^−2^ d^−1^ in the 40% treatment and 4.2 mol m^−2^ d^−1^ in the 60% treatment, the use of LED inter-lighting on cloudy days could supply plants with more than the minimum DLI requirement (Table 1). The light distribution among the middle and lower canopy leaves was greatly reduced with shading (Fig 2), but the inter-lighting improved it and DLI (Table 1), enhancing yield and fruit quality (Figs 5 and 6).

### Supplemental LED inter-lighting improved photosynthesis, growth, and yield when daily light integral is limiting

Both 40% and 60% shading treatments reduced the chlorophyll content of middle and lower leaves, and consequently reduced leaf photosynthetic rate (Figs 3 and 4). Leaf chlorophyll content is one of the most important factors determining photosynthetic rate [50, 51] and dry matter production [52], since the contribution of leaves to crop yield through photosynthetic assimilation relies on the amount of radiant energy absorbed by chlorophyll [22]. LED inter-lighting during cloudy days increased leaf chlorophyll content and photosynthetic rate in the mid-canopy leaves (Figs 3 and 4). It also significantly increased LMA but not LAI, indicating that it enhanced shoot dry mass production by improving leaf photosynthetic capacity (Table 2; Fig 5).

**Fig 7:**
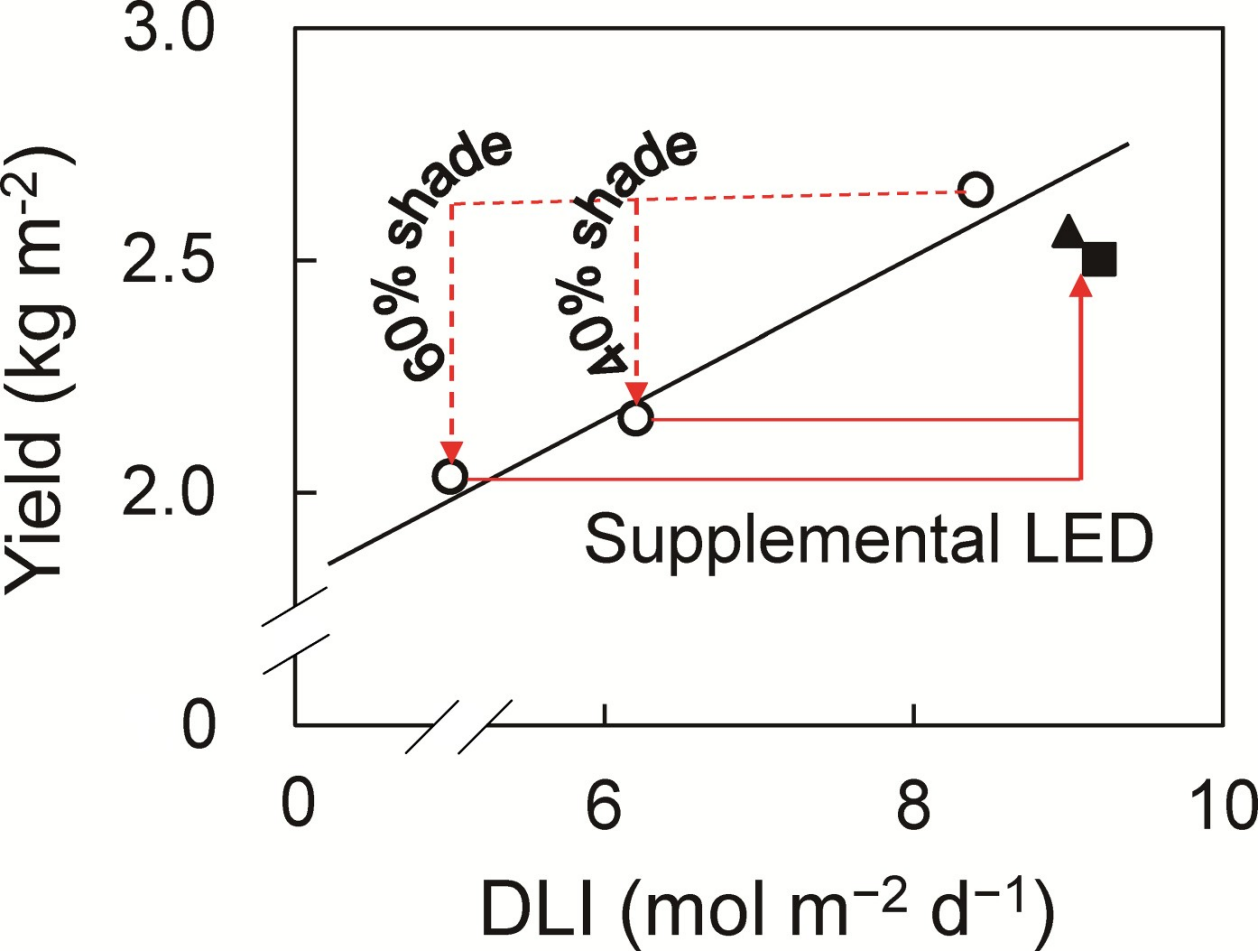
Relation between yield and daily light integral (DLI). Open circle: plants grown under shading during 0%, 40% and 60% of days, Filled triangle: plants grown under shading during 40% of days with supplemental LED inter-lighting, filled square: plants grown under shading during 60% of days with supplemental LED inter-lighting. The regression line among plants grown under shading during 0%, 40% and 60% of days is shown (y = 0.1869x + 1.0605, *R*^2^ = 0.97).

Generally, yield has an inverse relationship with shading level [11]. Cloud cover also decreases productivity and daily carbon gain owing to a dramatic reduction in total DLI [53]. Shading can decrease the soluble solids content and increase the titratable acidity of fruits [11]. In humid subtropical Brazil, 52% shading of tomato between September and December after anthesis reduced yield by 20% [54]. In our results, 30% shading during 40% and 60% of days reduced yield by 18.5% and 23.3% (Fig 5). This indicated that tomato yield has directly a proportional relationship with DLI (Fig 7), as a 1% reduction in DLI reduced yield by 0.7% in the 40% treatment and by 0.6% in the 60% treatment. Similarly, in previous work, a 1% reduction in DLI results in a 1% reduction in yield for greenhouse crops such as cucumber and sweet pepper throughout the cropping period [35]. The observed decreases in photosynthetic rate, growth, and yield of tomato with decreasing solar radiation are consistent with common understanding. However, our data clearly show that supplemental lighting improved DLI (Table 1) and vertical light distribution within the canopy (Fig 2), resulting in increases in leaf photosynthetic rate (Fig 4), growth and yield (Fig 5). In addition, supplemental LED inter-lighting compensated for reductions in total soluble solids content of fruits (Fig 6). It has been reported that developmental triggers and environmental signals, particularly light, influence ascorbic acid accumulation in leaves [55–57], however, the ascorbic acid concentration was not affected by the shading treatment in the present study. The ascorbic acid concentration might be affected not by DLI but by the maximum light intensities within a couple of days, since ascorbic acid can function as an antioxidant to alleviate the high-light stress for plants [58].

Previous studies showed that supplemental lighting improved canopy light interception, leaf photosynthetic capacity, assimilate supply to fruits, and crop productivity [8, 29, 30, 33]. However, some studies reported that it did not significantly increase yield of tomato and cucumber [12, 35, 59]. Part of the reason for the difference could be reduced vertical and horizontal light interception caused by extreme leaf curling, the LED-light spectrum used, or low irradiance [59]. Another reason could be that some tomato cultivars grow equally well whether lit from above or within [12]. Recently, it has been reported that daytime inter-lighting during summer improved mid-canopy light distribution but also increased temperature significantly [33], so high temperatures and high solar irradiation during midday in summer could exceed the optimal range for tomato production and thus reduce yield. Thus, both the growth season and the time of day when the light is applied could also determine the effectiveness of supplemental inter-lighting.

## Conclusion

Shading during both 40% and 60% of days significantly decreased DLI in the mid and lower canopy, resulting in reductions in tomato productivity. However, supplemental LED inter-lighting improved the light distribution within the plant profile and compensated for the DLI requirement of plants, improving plant growth and yield. These results clearly indicate that where cloudy or rainy days are frequent, supplemental LED inter-lighting could compensate for a shortage of light for plant growth and yield (both quantity and quality), allowing sustainable year-round tomato production.

## Supporting information

S1 Table Effects of LED inter-lighting on leaf photosynthetic capacity of single-truss tomato plants. Light-response curve was measured from 10:00 to 14:00 in representative leaves in three canopy layers in plants grownunder shading during 0%, 40% and 60% of days with or without LED inter-lighting. Data are means ± SEM (n = 5). Different letters indicated significant difference within the column (Tukey’s HSD test, *P* <0.05).
(PDF)
