# Supplementary material for: Supplemental LED inter-lighting compensates for a shortage of light for plant growth and yield under the lack of sunshine
Source: PLoS One. 2018 Nov 1;13(11):e0206592. doi: 10.1371/journal.pone.0206592 (PMC6211714; doi:10.1371/journal.pone.0206592)
Supplement: S1 Table — Light-response curve was measured from 10:00 to 14:00 in representative leaves in three canopy layers in plants grownunder shading during 0%, 40% and 60% of days with or without LED inter-lighting. Data are means ± SEM (n = 5). Different letters indicated significant difference within the column (Tukey’s HSD test, P <0.05). (PDF) [file pone.0206592.s001.pdf]

Supplemental LED inter-lighting compensates for a shortage of light for plant growth and yield under the lack of sunshine

Fasil Tadesse Tewolde, Kouta Shiina, Toru Maruo, Michiko Takagaki, Toyoki Kozai and Wataru Yamori*

Supplementary Table S1.

Effects of LED inter-lighting on leaf photosynthetic capacity of single-truss tomato plants. Light-response curve was measured from 10:00 to 14:00 in representative leaves in three canopy layers in plants grown under shading during 0%, 40% and 60% of days with or without LED inter-lighting. Data are means ± SEM (n = 5). Different letters indicated significant difference within the column (Tukey's HSD test, P < 0.05).

	(A) Top Canopy			(B) Mid Canopy			(A) Lower Canopy		
	Control	40% Shade	40% Shade & LED	Control	40% Shade	40% Shade & LED	Control	40% Shade	40% Shade & LED
0	-1.33 ± 0.18a	-1.42 ± 0.15a	-0.98 ± 0.11a	-0.76 ± 0.15a	-1.01 ± 0.23a	-1.19 ± 0.18a	-0.62 ± 0.21a	-0.55 ± 0.11a	-0.67 ± 0.16a
50	2.18 ± 0.11a	1.81 ± 0.11a	2.11 ± 0.09a	2.19 ± 0.13a	1.96 ± 0.15a	1.80 ± 0.11a	1.75 ± 0.11a	1.57 ± 0.21a	1.93 ± 0.14a
100	4.69 ± 0.09a	4.25 ± 0.07a	4.29 ± 0.12a	3.90 ± 0.18a	3.70 ± 0.21a	3.54 ± 0.12a	3.14 ± 0.23a	2.59 ± 0.35a	3.42 ± 0.09a
200	10.29 ± 0.25a	9.19 ± 0.16a	9.01 ± 0.35a	7.41 ± 0.38a	6.73 ± 0.55a	7.31 ± 0.26a	5.74 ± 0.51ab	4.37 ± 0.54b	6.30 ± 0.16a
400	12.51 ± 0.31a	10.96 ± 0.28a	10.92 ± 0.45a	8.43 ± 0.41a	7.69 ± 0.57a	8.84 ± 0.29a	6.57 ± 0.57ab	5.01 ± 0.60b	7.55 ± 0.30a
600	13.79 ± 0.37a	11.67 ± 0.34a	12.01 ± 0.63a	9.10 ± 0.41a	8.18 ± 0.67a	9.66 ± 0.37a	6.92 ± 0.68ab	5.35 ± 0.61b	8.25 ± 0.33a
900	14.35 ± 0.31a	12.25 ± 0.38b	12.69 ± 0.72a	9.34 ± 0.44b	8.40 ± 0.69b	10.32 ± 0.40a	7.14 ± 0.75ab	5.46 ± 0.67b	8.73 ± 0.40a
1000	14.81 ± 0.36a	12.59 ± 0.41a	13.13 ± 0.81a	9.61 ± 0.45ab	8.43 ± 0.62b	10.78 ± 0.45a	7.36 ± 0.74ab	5.55 ± 0.71b	8.99 ± 0.43a
1300	15.07 ± 0.33a	12.78 ± 0.50a	13.44 ± 0.84a	9.79 ± 0.54ab	8.64 ± 0.61b	11.02 ± 0.49a	7.36 ± 0.82ab	5.61 ± 0.68b	9.14 ± 0.44a
1500	15.35 ± 0.33a	13.04 ± 0.55a	13.71 ± 0.93a	10.10 ± 0.74ab	8.63 ± 0.61b	11.06 ± 0.50a	7.37 ± 0.73ab	5.67 ± 0.64b	9.21 ± 0.46a
	(A) Top Canopy			(B) Mid Canopy			(A) Lower Canopy		
	Control	60% Shade	60% Shade & LED	Control	60% Shade	60% Shade & LED	Control	60% Shade	60% Shade & LED
0	-1.33 ± 0.18a	-1.15 ± 0.19a	-1.27 ± 0.10a	-0.76 ± 0.15a	-0.60 ± 0.22a	-0.83 ± 0.13a	-0.62 ± 0.21a	-0.31 ± 0.06a	-1.07 ± 0.15a
50	2.18 ± 0.11a	2.21 ± 0.19a	2.01 ± 0.16a	2.19 ± 0.13a	1.74 ± 0.11a	2.22 ± 0.19a	1.75 ± 0.11a	1.46 ± 0.13a	1.70 ± 0.23a
100	4.69 ± 0.09a	4.23 ± 0.24a	4.33 ± 0.22a	3.90 ± 0.18a	3.05 ± 0.08b	4.43 ± 0.29a	3.14 ± 0.23a	2.48 ± 0.24a	3.38 ± 0.32a
200	10.29 ± 0.25a	9.01 ± 0.26a	9.19 ± 0.57a	7.41 ± 0.38b	5.63 ± 0.17c	9.01 ± 0.52a	5.74 ± 0.51ab	4.01 ± 0.32b	6.68 ± 0.69a
400	12.51 ± 0.31a	10.64 ± 0.39b	11.15 ± 0.59a	8.43 ± 0.41b	6.61 ± 0.29c	10.71 ± 0.56a	6.57 ± 0.57a	4.20 ± 0.29b	8.04 ± 0.71a
600	13.79 ± 0.37a	11.65 ± 0.51b	12.11 ± 0.67a	9.10 ± 0.41b	7.15 ± 0.39b	11.51 ± 0.54a	6.92 ± 0.68a	4.44 ± 0.33b	8.73 ± 0.66a
900	14.35 ± 0.31a	12.17 ± 0.56b	12.61 ± 0.64a	9.34 ± 0.44b	7.41 ± 0.42b	12.01 ± 0.64a	7.14 ± 0.75a	4.49 ± 0.35b	9.06 ± 0.75a
1000	14.81 ± 0.36a	12.52 ± 0.62a	13.08 ± 0.55a	9.61 ± 0.45b	7.51 ± 0.39c	12.17 ± 0.58a	7.36 ± 0.74a	4.59 ± 0.27b	9.32 ± 0.68a
1300	15.07 ± 0.33a	12.81 ± 0.61a	13.23 ± 0.63a	9.79 ± 0.54b	7.55 ± 0.45c	12.32 ± 0.53a	7.36 ± 0.82a	4.63 ± 0.34b	9.45 ± 0.67a
1500	15.35 ± 0.33a	13.01 ± 0.67a	13.28 ± 0.56a	10.10 ± 0.74a	7.67 ± 0.42c	12.35 ± 0.54a	7.37 ± 0.73a	4.71 ± 0.31b	9.40 ± 0.71a
